# The role of microglial P2X7: modulation of cell death and cytokine release

**DOI:** 10.1186/s12974-017-0904-8

**Published:** 2017-07-17

**Authors:** Yingbo He, Natalie Taylor, Lawrence Fourgeaud, Anindya Bhattacharya

**Affiliations:** Janssen Research & Development, LLC., Neuroscience Drug Discovery, 3210 Merryfield Row, San Diego, CA 92121 USA

**Keywords:** Microglia, P2X7, Neuroinflammation, Cell death, Cytokine

## Abstract

**Background:**

ATP-gated P2X7 is a non-selective cation channel, which participates in a wide range of cellular functions as well as pathophysiological processes including neuropathic pain, immune response, and neuroinflammation. Despite its abundant expression in microglia, the role of P2X7 in neuroinflammation still remains unclear.

**Methods:**

Primary microglia were isolated from cortices of P0-2 C57BL/6 wild-type or P2X7 knockout (P2X7^−/−^) mouse pups. Lipopolysaccharide, lipopolysaccharide plus IFNγ, or IL4 plus IL13 were used to polarize microglia to pro-inflammatory or anti-inflammatory states. *P2rx7* expression level in resting or activated mouse and human microglia was measured by RNA-sequencing and quantitative real-time PCR. Microglial cell death was measured by cell counting kit-8 and immunocytochemistry, and microglial secretion in wild-type or P2X7^−/−^ microglia was examined by Luminex multiplex assay or ELISA using P2X7 agonist BzATP or P2X7 antagonist A-804598. P2X7 signaling was analyzed by Western blot.

**Results:**

First, we confirmed that *P2rx7* is constitutively expressed in mouse and human primary microglia. Moreover, *P2rx7* mRNA level was downregulated in mouse microglia under both pro- and anti-inflammatory conditions. Second, P2X7 agonist BzATP caused cell death of mouse microglia, while this effect was suppressed either by P2X7 knockout or by A-804598 under both basal and pro-inflammatory conditions, which suggests the mediating role of P2X7 in BzATP-induced microglial cell death. Third, BzATP-induced release of IL1 family cytokines including IL1α, IL1β, and IL18 was blocked in P2X7^−/−^ microglia or by A-804598 in pro-inflammatory microglia, while the release of other cytokines/chemokines was independent of P2X7 activation. These findings support the specific role of P2X7 in IL1 family cytokine release. Finally, P2X7 activation was discovered to be linked to AKT and ERK pathways, which may be the underlying mechanism of P2X7 functions in microglia.

**Conclusions:**

These results reveal that P2X7 mediates BzATP-induced microglial cell death and specific release of IL1 family cytokines, indicating the important role of P2X7 in neuroinflammation and implying the potential of targeting P2X7 for the treatment of neuroinflammatory disorders.

**Electronic supplementary material:**

The online version of this article (doi:10.1186/s12974-017-0904-8) contains supplementary material, which is available to authorized users.

## Background

Growing evidence supports that neuroinflammation is involved in pathogenesis of a variety of neurological disorders, including Alzheimer’s disease, Parkinson’s disease, and psychiatric diseases [1, 2]. Therefore, dampening neuroinflammation has been considered one of the leading therapeutic strategies for such diseases. Microglia, the resident immune cells of the brain parenchyma, play critical roles in neuroinflammation [3, 4]. At quiescent state, microglia display characteristically ramified morphology with numerous branching processes, which dynamically survey the brain microenvironment [5]. In response to injury, microglia become activated with amoeboid morphology by retracting their ramified processes and produce inflammatory cytokines such as tumor necrosis factor α (TNFα), interleukin (IL) 6, and IL1β [6]. However, the molecular mechanisms underlying microglial activation have not been fully clarified and need to be further explored.

P2X7 is an ATP-gated, nonselective cation channel allowing Ca^2+^ and Na^+^ influx and K^+^ efflux [7]. In the periphery, P2X7 is abundant in hematopoietic lineage cells, including mast cells, B and T lymphocytes, monocytes, and macrophages [8]. In the brain, P2X7 is expressed abundantly in microglia, while its expression and function in neurons and astrocytes is debatable [9–14]. Additionally, there is a conflict with the expression of P2X7 between quiescent and activated microglia. Choi et al. reported that exposure of cultured human microglia to lipopolysaccharide (LPS) increases the expression of P2X7 in a time-dependent manner [15], whereas some microglial cell lines treated with LPS showed a decreased P2X7 level [16]. Therefore, the detailed mechanism for regulating P2X7 expression in mouse primary microglia under pro-inflammatory and anti-inflammatory conditions remains uncertain.

Functionally, P2X7 is best known for promoting NLRP3 inflammasome assembly and caspase-1-dependent release of proinflammatory IL1β and IL18 from innate immune cells after exposure to LPS and ATP [17–19]. In activated macrophages, beyond NLRP3-inflammasome induced IL1β and IL18 release, P2X7 also controls the release of other proteins, including TNFα and chemokine (C-C motif) ligand 2 (CCL2) [20], which suggests that P2X7 is coupled to a secretome in macrophages. However, whether or not this is also the case in activated microglia has not been well characterized yet.

Another hallmark of P2X7 activation is the formation of plasma membrane pores permeable to molecules up to a molecular weight of 900 Da with prolonged exposure to ATP, which leads to dramatic elevation of intracellular Ca^2+^, depletion of intracellular ions and metabolites, and ultimately cell death [21]. P2X7-mediated cell death has been reported in several types of cells, such as macrophages [22], leukemic cells [23], and rat microglial cell line N9 and N13 [24]. In contrast, recently, it has been shown that P2X7 plays a trophic role in supporting cell growth and proliferation. Overexpression of P2X7 evokes both microglia activation and proliferation [25]. P2X7 is also required for embryonic microglial proliferation, because absence of P2X7 leads to a decreased microglia density [26]. This contradiction highlights the need to systemically investigate P2X7 functions in primary microglia.

To this end, we examined the expression and modulation of P2X7 in mouse and human microglia by RNA-sequencing (RNA-seq) and quantitative real-time PCR. Using genetic and pharmacological approaches, we also systemically investigated the role of P2X7 in microglial survival and activation, as well as the underlying signaling cascades. The results would extend our understanding of P2X7 in microglia and provide new insights in the mechanisms of P2X7 in neuroinflammation.

## Methods

### Animals

Wild-type (WT) C57BL/6 pregnant mice were obtained from Charles River Laboratories, Inc. P2X7^−/−^ mice used in this study were derived from Pfizer. P2X7^−/−^ pregnant mice and their background and age-matched WT pregnant mice were received from The Jackson Laboratory. Mice were allowed to acclimate for 7 days after receipt. They were kept on a 12-h light/dark cycle and allowed free access to food and water. All animal care and use complied with the Guide for the Care and Use of Laboratory.

### Reagents

3′-O-(4-Benzoyl) benzoyl adenosine 5′-triphosphate (BzATP) and LPS were purchased from Sigma-Aldrich. Interferon γ (IFNγ), TNFα, IL6, and IL1β were purchased from Biolegend. P2X7 antagonist A-804598, ERK inhibitor U0126, and AKT inhibitor LY294002 were obtained from Tocris.

### Cell culture

Cortices from P0-2 C57BL/6 mouse pups were dissected and stripped of meninges and mechanically dissociated with a hand homogenizer and a 25-gauge needle. The cell suspension was seeded into poly-l-lysine-coated (Sigma-Aldrich) T150 tissue culture flasks and maintained in DMEM/F12 with 10% FBS and 1% penicillin-streptomycin for 10–14 days to grow a confluent mixed astrocyte/microglia population. We collected and applied the cells to an antigen-antibody-mediated magnetic cell-sorting (MACS, Miltenyi Biotech) assay to positively select microglia. The mixed glial population was re-suspended in MACS buffer (Miltenyi Biotech) and incubated with CD11b MicroBeads (Miltenyi Biotech). The cell suspension was then applied to LS separation column (Miltenyi Biotech) fitted into a QuadroMACS cell separator (Miltenyi Biotech). Unlabeled cells were allowed to pass through the column while labeled cells remained captured in the magnetic field. After washing the column with MACS buffer, the column was then removed from the magnetic separator and flushed with MACS buffer to collect the purified microglia population. For an increased level of purity, the eluted microglia population was passed through a new LS separation column a second time. The purity of microglia used in our study was more than 95% assessed by immunocytochemistry (data not shown).

Human primary microglia (Catalog #1900) and astrocytes (Catalog #1800) were obtained from ScienCell Research Laboratories, Inc., and cultured as instructed.

### RNA extraction, reverse transcription PCR, and quantitative real-time PCR

Microglia or brain tissues were homogenized, and total RNA was extracted using RNeasy plus mini kit (Qiagen). Total RNA concentrations were measured using NanoDrop ND-1000 spectrophotometer. For RNA-seq, RNA quality was assessed by using Agilent RNA 6000 Nano Kit and Agilent 2100 Bioanalyzer according to the manufacturer’s instructions before sequencing by BGI, a fee-for-service provider. For other experiments, RNA was reverse-transcribed into cDNA using Superscript III reverse transcriptase (Invitrogen) with random hexamer primers. Transcript abundance was determined by quantitative PCR using SYBR Green PCR mix (Applied Biosystems), with primer pairs against *P2rx7* and *Gapdh*. Three *P2rx7* spliced variants were amplified by PCR with corresponding primers, and the PCR products were separated by electrophoresis on a 1.5% agarose gel. The following primer pairs were used for quantitative real-time PCR:

- *Gapdh*: 5′ AGGTCGGTGTGAACGGATTTG 3′ (F) and 5′ TGTAGACCATGTAGTTGAGGTCA 3′ (R)
- *P2rx7*: 5′ GACAAACAAAGTCACCCGGAT 3′ (F) and 5′ CGCTCACCAAAGCAAAGCTAAT 3′ (R)

Primers for reverse transcription PCR:

- *P2rx7a*: 5′ TCAGTAGGGATACTTGAAGCC 3′ (R)
- *P2rx7b*: 5′ TCTGTGAGAAACAAGTATCTAGGTTGG 3′ (R)
- *P2rx7c*: 5′ TCAGGTGCGCATACATACATG 3′ (R)
- *Gapdh*: 5′ TCCACCCATGGCAAATTCCATG 3′ (F) and 5′ TGGACTCCACGACGTACTCAGC 3′ (R)

Forward primer shared by *P2rx7* variants is 5′ TGCTCTTCTGACCGGCGTTG 3′ (F)

### Immunocytochemistry and immunohistochemstry

Immunocytochemistry was performed as described previously [27]. Briefly, cells were fixed with 4% paraformaldehyde and permeabilized by 0.1% Triton X-100. After blocking with 10% donkey serum, fixed cells were incubated with primary antibodies (Iba1, 1:1,000, WAKO Chemicals; GFAP, 1:1,000, Abcam) for 2 h followed by fluorochrome-conjugated secondary antibodies (Alexa Fluor 488 and 555, 1:200, Molecular Probes, respectively). Nuclei were counterstained with DAPI. Fluorescence images were acquired using a confocal-laser microscope (LSM 700; Carl Zeiss MicroImaging) with a multi-track configuration.

For immunohistochemistry, WT and P2X7^−/−^ aged matched mice were perfused. Brains were dissected out, cryo-protected, and cut. Brain sections were stained with primary antibodies (P2X7, 1:500, Sigma; Iba1, 1:500, Abcam; GFAP, 1:500, Abcam) for 48 h at 4 °C followed by fluorochrome-conjugated secondary antibodies (Alexa Fluor 488, 647, and Cy3, 1:500, Jackson Laboratory, respectively). Nuclei were counterstained with Hoechst. Images were acquired using a confocal-laser microscope (LSM 700; Carl Zeiss MicroImaging) and displayed with maximum projection of z-stacks.

### Enzyme-linked immunosorbent assay (ELISA) and secretome analysis

ELISA kits for mouse IL1β and IL18 (R&D systems) were used for quantification of IL1β and IL18 in cell culture supernatants following the manufacturer’s instruction.

The relative concentrations of secreted molecules in cell supernatants were measured using antibody-based 38-plex immunoassays (Luminex, R&D systems). The 38 secreted proteins we measured were as follows: CCL2/JE/MCP1, CCL3/MIP1α, CCL4/MIP1β, CCL5/RANTES, CCL20/MIP3α, CXCL1/KC, CXCL2/MIP2, CXCL10/IP10/CRG2, CXCL12/SDF1α, FGFb, FGF21, GCSF, GMCSF, IFNγ, IGFI, IL1α, IL1β, IL2, IL4, IL5, IL6, IL10, IL12 p70, IL13, IL17A, IL23 p19, IL33, LIX, MCSF, MMP9, Resistin, TNFα, VEGF, CCL11/Eotaxin, CCL22/MDC, CXCL9/MIG, IL9, and RAGE. To generate proteomic heat maps, we normalized immunoassay measurements of the listed proteins and clustered them using an unsupervised clustering algorithm (Array Studio). Any undetectable proteins for a sample were removed from the analysis.

### Cytotoxicity assay

Cell viability was determined by cell counting kit-8 (CCK-8, Dojindo), which measures mitochondrial dehydrogenase activity inside the cells. Briefly, 10 μl of CCK-8 solution was added to 100 μl of media in each well of the plate. After incubating the plate for 2–4 h at 37 °C, the absorbance at 450 nm was measured using the Bio-Rad microplate reader.

### Western blots

Cells were homogenized and lysed using RIPA buffer (Amresco) with protease and phosphatase inhibitors (Sigma and Roche, respectively). After centrifugation at 13,000*g*, protein concentrations were measured using the BCA protein assay kit (Pierce) and lysates were separated on a 4–12% Bis-Tris gels (Invitrogen) using MOPS sodium dodecyl sulfate running buffer (Invitrogen). Proteins were transferred with the iBlot system onto nitrocellulose membranes (Novex) and incubated with antibodies p-AKT (1:1000, Cell Signaling Technology), p-ERK (1:1000, Cell Signaling Technology), AKT (1:1000, Cell Signaling Technology), ERK (1:1000, Cell Signaling Technology), and GAPDH (1:1000, Millipore). Signal intensities were detected using ECL western blotting detection reagents (Amersham Biosciences) and evaluated by ImageJ.

### Statistical analysis

Data were statistically compared using one-way or two-way ANOVA followed by Tukey’s or Dunnett’s post hoc test among multiple groups using GraphPad Prism 6 (GraphPad Software, Inc.). *P* < 0.05 was considered statistically significant.

## Results

### Expression of P2X7 in primary microglia

**Fig. 1:**
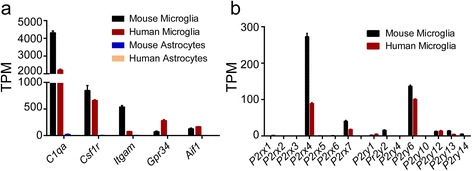
P2RX and P2RY family expression in microglia. **a** mRNA levels of microglial marker genes were measured by RNA-seq in human and mouse microglia and astrocytes. **b** mRNA levels of seven *P2rx* subtypes and eight *P2ry* subtypes were measured by RNA-seq in both human and mouse microglia. *TPM*, transcripts per kilobase million. Data are shown as mean + SD, *n* = 4

P2X and P2Y purinergic receptor families play critical roles in neuropathic pain and neuroinflammation. In order to determine their functions in various cell types in the brain, we first used RNA-seq to identify their expression in primary mouse and human microglia. As expected, microglial marker genes such as *C1qa*, *Csf1r*, *Itgam*, *Gpr34*, and *Aif1*, were highly expressed in mouse and human microglia but not in astrocytes, thus demonstrating the specificity of microglia used in this study (Fig. 1a). Additionally, among all the P2X and P2Y subtypes, *P2rx4*, *P2rx7*, and *P2ry6* exhibited robust constitutive expression in both primary mouse and human microglia (Fig. 1b). Particularly, *P2rx7* showed a nearly threefold higher mRNA level in mouse microglia than in human microglia, and more importantly, mouse microglia are much easier to access; we therefore aimed to examine its expression and function in mouse microglia under pro- and anti-inflammatory conditions.

### P2X7 expression is regulated in microglia

**Fig. 2:**
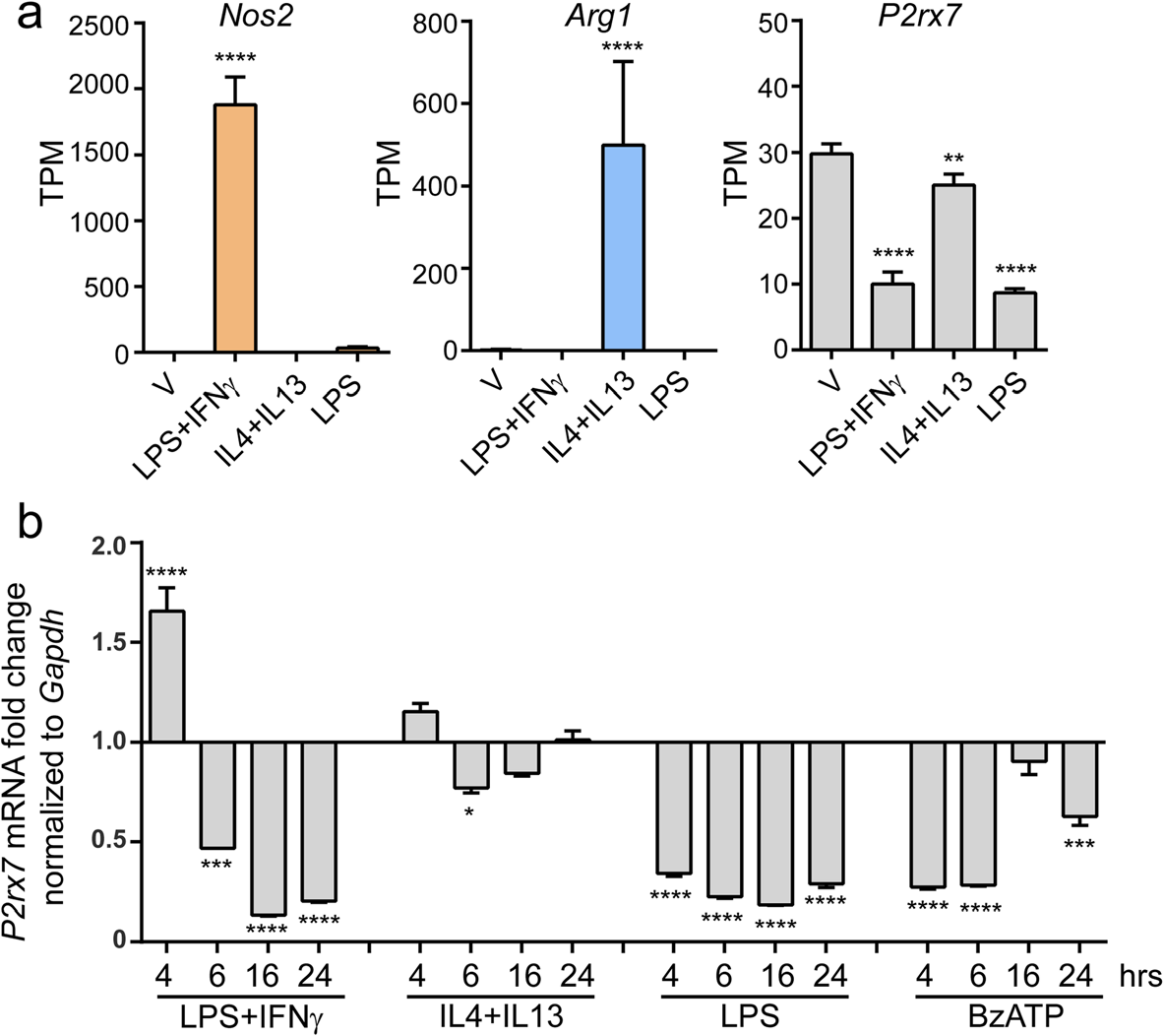
Regulation of P2X7 expression in microglia. **a** mRNA levels of *Nos2*, *Arg1*, and *P2rx7* in mouse microglia under indicated conditions (100 ng/ml LPS plus 10 ng/ml IFNγ, 10 ng/ml IL4 plus 10 ng/ml IL13, or 100 ng/ml LPS) for 24 h were evaluated by RNA-seq. Data are shown as mean + SD, *n* = 4. **b** *P2rx7* mRNA levels in mouse microglia exposed to 100 ng/ml LPS plus 10 ng/ml IFNγ, 10 ng/ml IL4 plus 10 ng/ml IL13, 100 ng/ml LPS, or 380 μM BzATP at indicated time durations were measured by quantitative real-time PCR. *P2rx7* mRNA level was normalized to *Gapdh* and presented as fold change compared to vehicle. All experiments were carried out in triplicate and repeated twice independently. Statistical analysis was performed by comparing each condition with vehicle. One-way ANOVA followed by Dunnett’s post hoc test. *P < 0.05; **P < 0.01; ***P < 0.001; ****P < 0.0001

To monitor the modulation of *P2rx7* mRNA in microglia, we polarized mouse microglia to either a pro-inflammatory state (LPS plus IFNγ or LPS alone) or an anti-inflammatory state (IL4 plus IL13) for 24 h and used RNA-seq to measure the transcript level. Indeed, mRNA level of a pro-inflammatory gene *nitric oxide synthase 2* (*Nos2*) was induced almost 2000-fold higher by LPS plus IFNγ as compared with vehicle-treated control, whereas IL4 plus IL13 stimulated expression of anti-inflammatory gene *arginase 1* (*Arg1*) with up to more than 400-fold increase (Fig. 2a). To our surprise, *P2rx7* mRNA level was reduced by almost 70% in cells treated either with LPS plus IFNγ or LPS alone compared to that in vehicle-treated cells. In addition, treatment with IL4 plus IL13 resulted in an approximate 10% decrease in *P2rx7* mRNA level (Fig. 2a).

To further confirm the RNA-seq results and test whether the regulation of *P2rx7* mRNA is time-dependent, we treated mouse microglia with the same stimuli for various time periods and then performed quantitative real-time PCR on collected RNA. Interestingly, as depicted in Fig. 2b, *P2rx7* mRNA level in LPS plus IFNγ-stimulated cells transiently increased 60% at the first 4 h and then decreased more than 50% at 6, 16, and 24 h. A similar but lesser trend was also observed in cells treated with IL4 plus IL13 (Fig. 2b). Moreover, *P2rx7* mRNA levels were decreased more than half at all-time points upon LPS treatment (Fig. 2b). Additionally, P2X7 agonist BzATP also suppressed *P2rx7* expression at most of the time points (Fig. 2b). However, these changes in *P2rx7* mRNA expression were modest compared to *Nos2* and *Arg1* in Fig. 2a. Altogether, the data indicate that *P2rx7* mRNA is constitutively expressed in microglia and most likely to be regulated in both pro- and anti-inflammatory states.

### Activation of P2X7 mediates cell death in microglia

**Fig. 3:**
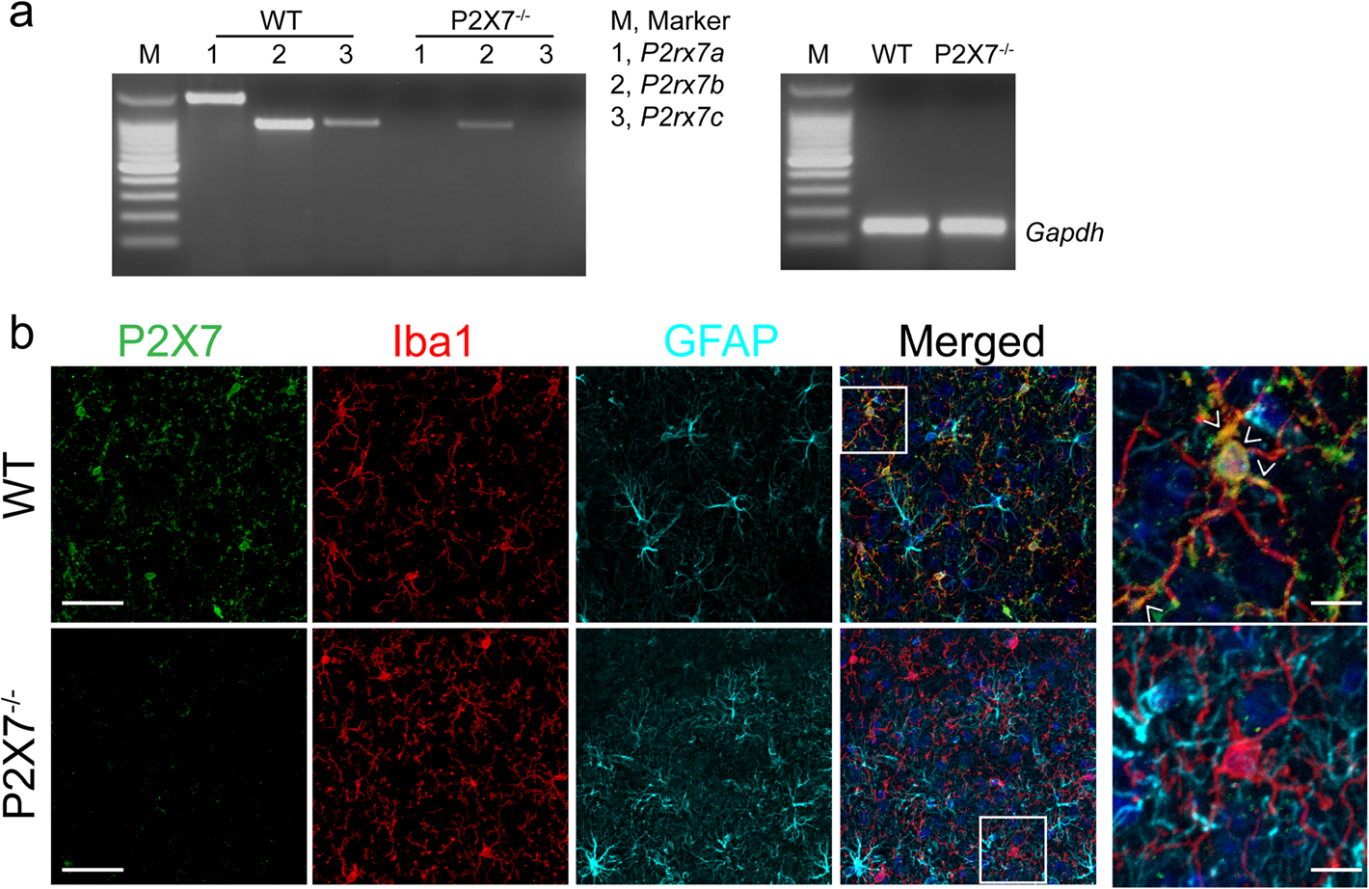
P2X7 expression in P2X7^−/−^ mouse brain. **a** Representative images of gel analysis of reverse transcription PCR products for *P2rx7* spliced variants from WT and P2X7^−/−^ microglia. DNA ladder is at 100 bp intervals. *Gapdh* was used as an internal loading control. **b** Representative images of immunofluorescence for P2X7 (*green*), Iba1 (*red*), and GFAP (*cyan*) in the cortex of WT and P2X7^−/−^ mice. Nuclei were counterstained with Hoechst (*blue*). Selected *white boxes* were zoomed in right panels. *Arrowhead* indicates representative colocalization of P2X7 and Iba1. Scale bar, 50 μm and 10 μm (zoomed images)

P2X7 mediates ATP-induced cell death in different cell types including macrophages and T lymphocytes [22, 23]. To investigate the role of P2X7 in guiding microglial fate, we used P2X7^−/−^ mouse line to test whether P2X7 is necessary for ATP-induced microglial cell death. Mouse *P2rx7* transcript has been identified as three splice variants of *P2rx7a*, *P2rx7b*, and *P2rx7c* distinguished by different C-terminus lengths [28]. Initially, we analyzed the expression of the three variants in microglia derived from WT and P2X7^−/−^ mouse with C57BL/6 background by reverse transcription PCR. Results showed that the three variants could be detected in WT microglia, among which variant *P2rx7a* is the predominant form (Fig. 3a). Nevertheless, for P2X7^−/−^ microglia, neither *P2rx7a* nor *P2rx7c* could be detected; while *P2rx7b* showed a trace expression level (Fig. 3a). This finding was consistent with previous report [28]. Additionally, we examined P2X7 protein expression in the cortex of WT and P2X7^−/−^ mice by immunohistochemistry. In agreement with our gene expression analysis, P2X7 was indeed expressed in the brain of WT mouse and dominantly colocalized with microglial marker Iba1 (Fig. 3b). In contrast, there was no specific P2X7 signal colocalized with Iba1 in the cortex of P2X7^−/−^ mice (Fig. 3b). Collectively, these data demonstrated that P2X7 is knocked down in the brain of P2X7^−/−^ mice.

**Fig. 4:**
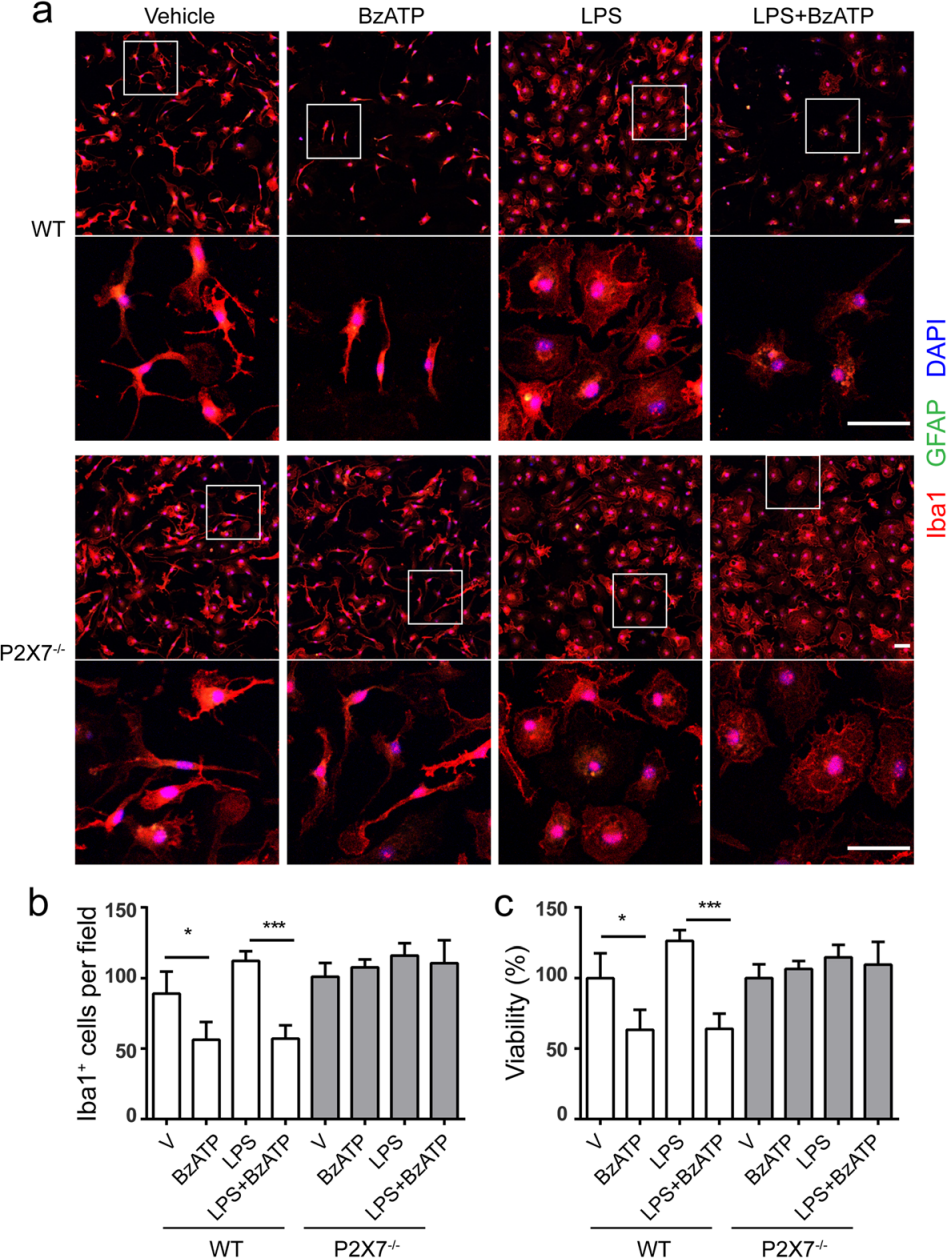
P2X7 is necessary for BzATP-induced microglial cell death. Microglia from WT and P2X7^−/−^ mice were primed with or without 100 ng/ml LPS for 22 h following treatment of 380 μM BzATP for additional 4.5 h. **a** Representative images of double immunofluorescence for GFAP (*green*) and Iba1 (*red*) under indicated conditions. Nuclei were counterstained with DAPI (*blue*). *White box* indicates position of high magnification images shown below. Scale bar, 50 μm. **b** Iba1^+^ microglia quantified by counting at least 3 randomly selected fields under each condition. **c** Cell viability was calculated by normalizing to vehicle-treated control. Data are shown as mean + SD, *n* = 3. Experiment was repeated twice independently. One-way ANOVA followed by Tukey’s post hoc test. *P < 0.05; ***P < 0.001

Next, we investigated the necessity of P2X7 in ATP-induced microglial cell death. BzATP was used as an agonist of P2X7 because it is approximately tenfold more potent and more stable than ATP. Concentration response data indicated that BzATP significantly induced microglial cell death starting at the concentration of 380 μM (see Additional file 1). Hence, primary microglia isolated from the brains of postnatal WT or P2X7^−/−^ mouse were primed with or without 100 ng/ml LPS prior to exposure to 380 μM BzATP. Microglial morphology was examined by confocal microscopy, and cell number was counted manually. As presented in Fig. 4a, under normal growth conditions, WT and P2X7^−/−^ microglia displayed ramified cell morphology. Nevertheless, when being treated with BzATP, WT cells showed shorter processes and a significant decrease (nearly 50%) in cell number as compared to untreated control, and this phenotype was not observed in P2X7^−/−^ microglia (Fig. 4a) suggesting a role of P2X7 in microglial cell death. In addition, treatment with LPS induced morphological changes from ramified to amoeboid and proliferation in both sets of cells (Fig. 4a) indicating P2X7 is not involved in these processes in activated microglia. When exposed to LPS plus BzATP, however, WT microglia exhibited morphological changes and a considerable reduction in cell number, whereas P2X7^−/−^ microglia only showed morphological changes (Fig. 4a–c). Taken together, these results demonstrate that P2X7 mediates BzATP-induced cell death but not LPS-induced morphological changes in both inactive and activated microglia.

### Knockout of P2X7 inhibits BzATP-induced release of IL1β and IL18

**Fig. 5:**
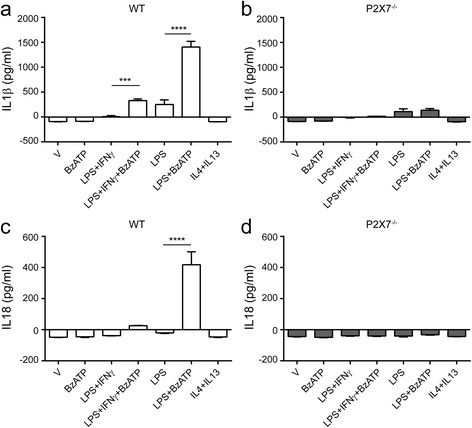
Cytokine secretion by WT and P2X7^−/−^ microglia under pro- and anti-inflammatory conditions. Microglia from WT and P2X7^−/−^ mice were treated with 100 ng/ml LPS, 100 ng/ml LPS plus 10 ng/ml IFNγ, or 10 ng/ml IL4 plus 10 ng/ml IL13 for 22 h in the presence or absence of 380 μM BzATP for additional 2 h. **a**–**d** The secretion of IL1β from WT microglia (**a**) and P2X7^−/−^ microglia (**b**), as well as IL18 release from WT microglia (**c**) and P2X7^−/−^ microglia (**d**) was assessed by ELISA. Data are shown as mean + SD of triplicates. Experiment repeated twice independently. One-way ANOVA followed by Tukey’s post hoc test. ***P < 0.001; ****P < 0.0001

P2X7 plays a key role in production and release of IL1β and IL18 in immune cells. To examine whether P2X7 has such a function in microglia, LPS or LPS plus IFNγ-primed WT and P2X7^−/−^ microglia were treated with BzATP and the secretion of inflammatory cytokines IL1β and IL18 was quantified by ELISA. Based on the outcomes of concentration- and time-dependent responses (see Additional file 2), we chose 380 μM BzATP to treat microglia for 2 h. As shown in Fig. 5a, c, in LPS-primed WT microglia, BzATP elicited a significant increase in the secretion of IL1β and IL18 as compared to vehicle-treated control, which is consistent with previous reports [17]. However, BzATP-induced release of IL1β and IL18 was completely abolished in microglia cultured from P2X7^−/−^ mice (Fig. 5b, d). Interestingly, LPS alone-induced release of IL1β in WT microglia was suppressed when microglia were co-stimulated with LPS plus IFNγ. In addition, BzATP alone and anti-inflammatory cytokines of IL4 plus IL13 did not induce IL1β and IL18 release either in WT or P2X7^−/−^ microglia (Fig. 5a–d). Taken together, these data demonstrate that in microglia, P2X7 modulates the production of IL1β and IL18 in response of BzATP in a LPS-dependent manner.

### P2X7 antagonist protects microglia against ATP-induced cytotoxicity

**Fig. 6:**
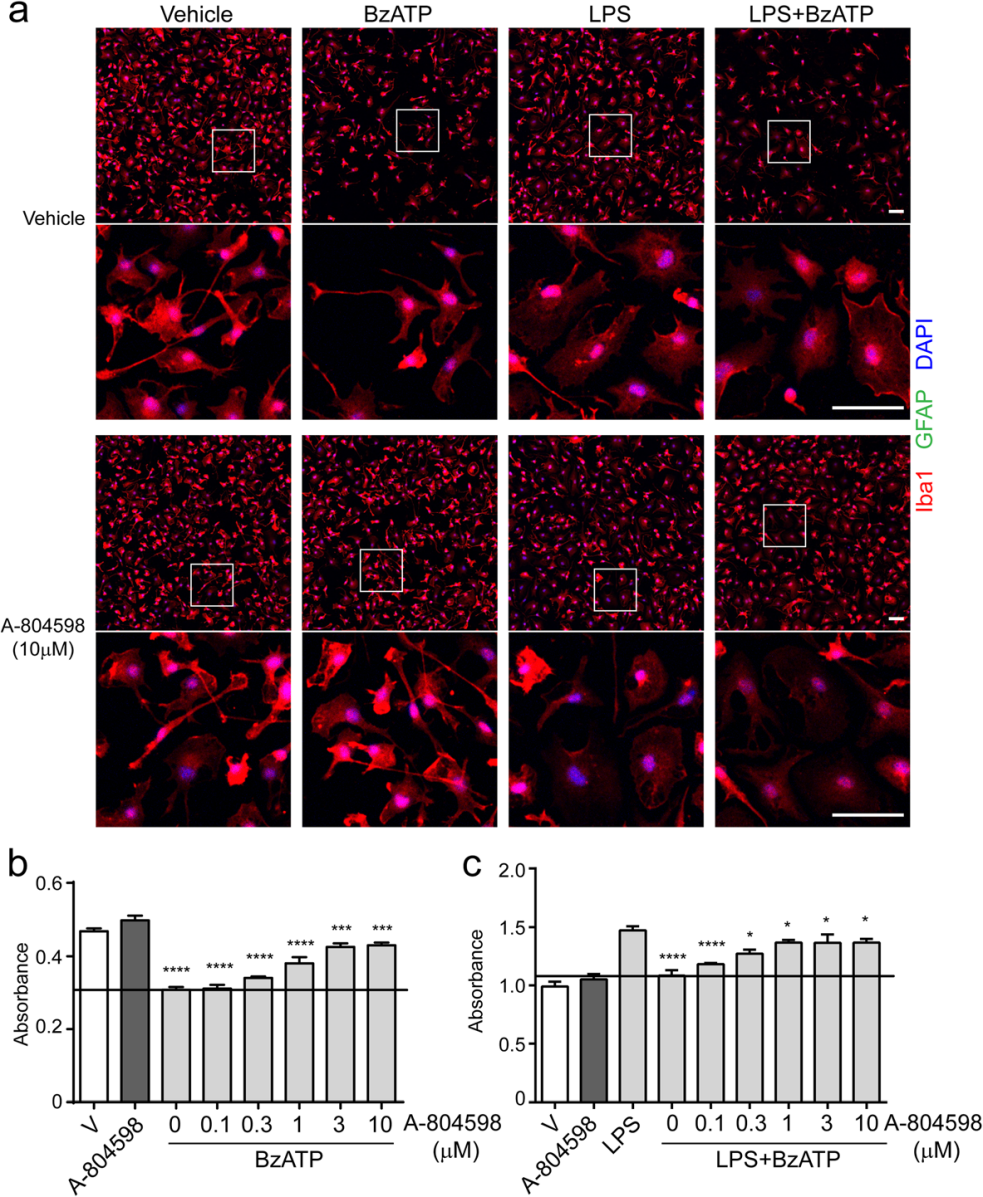
P2X7 antagonist attenuates BzATP-induced microglial cell death. Microglia were pre-treated with different concentrations of P2X7 antagonist A-804598 for 1 h before exposure to 100 ng/ml LPS for 22 h in the absence or presence of 380 μM BzATP for additional 4.5 h. **a** Immunocytochemical images of microglia with or without P2X7 antagonist treatment under conditions of BzATP alone, LPS alone, or LPS plus BzATP. *White box* indicates position of high magnification images shown below. Iba1 as microglial marker shown in *red*, GFAP as astrocyte marker shown in *green*, DAPI stains nuclei. Scale bar, 50 μm. **b** The number of microglia treated by BzATP after pre-treatment with different concentrations of A-804598 quantified by CCK-8 kit. **c** The number of LPS-primed microglia after pre-treatment with different concentrations of A-804598 in the presence of BzATP quantified by CCK-8 kit. Data are shown as mean + SD. Experiment was done in six replicates and repeated three times independently. Statistical analysis was performed between vehicle- and BzATP-treated groups in **b**, between LPS- and BzATP-treated groups in **c**. One-way ANOVA followed by Tukey’s post hoc test. *P < 0.05; ***P < 0.001; ****P < 0.0001

To complement the results of P2X7 knockdown, we sought to test the effect of a P2X7 antagonist (A-804598) in murine microglia. Primary microglia were incubated with varying concentrations of A-804598 for 1 h prior to exposure to BzATP. Cell viability was measured by CCK-8 assay, and microglial morphology was examined by confocal microscopy. As shown in Fig. 6a, BzATP alone resulted in approximately 40% microglial cell loss, while pre-incubation with A-804598 significantly attenuated BzATP-induced cell loss in a concentration-dependent manner. Three micromolar A-804598 exhibited the greatest protective effect against BzATP-induced cytotoxicity (Fig. 6b). Furthermore, we validated the protective effect of A-804598 in activated microglia. LPS-primed microglia were first exposed to varying concentrations of A-804598 and then treated with BzATP. As expected, the primed microglia exhibited amoeboid morphology and significant cell loss in response to BzATP (Fig. 6a). However, results of confocal microscopy and CCK-8 assay showed that cell loss by BzATP were counteracted by pre-incubation with A-804598 concentration dependently (Fig. 6c). Taken together, our findings revealed the protective effects of P2X7 antagonist A-804598 against BzATP-induced cytotoxicity in both inactivated and activated microglia, further demonstrating the mediating role of P2X7 in ATP-induced microglial cell death.

### P2X7 antagonist significantly attenuates ATP-induced release of IL1α and IL1β in microglia

**Fig. 7:**
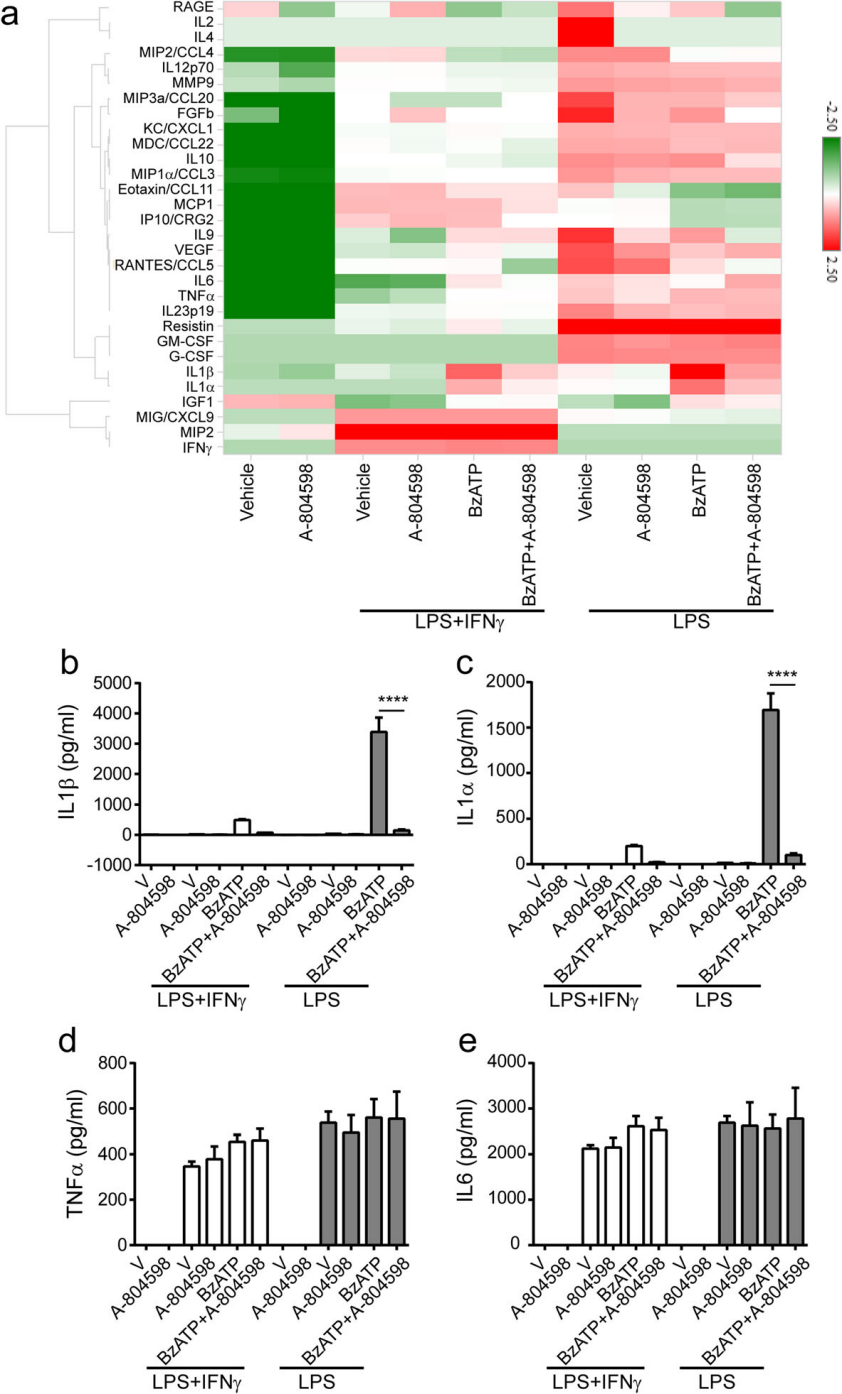
Secretome of microglia treated with P2X7 antagonist upon pro-inflammatory stimulation. Microglia were incubated with 1 μM P2X7 antagonist A-804598 for an hour before exposure to 100 ng/ml LPS or 100 ng/ml LPS plus 10 ng/ml IFNγ for 22 h in the presence or absence of 380 μM BzATP for additional 2 h. **a** Heat map and hierarchical clustering of detectable 30 proteins released from microglia measured by Luminex multiplex. **b**–**e** Histogram of IL1β (**b**), IL1α (**c**), TNFα (**d**), and IL6 (**e**) release obtained from **a**. Data are shown as mean + SD of triplicates. Experiment repeated twice independently. One-way ANOVA followed by Tukey’s post hoc test. ****P < 0.0001

P2X7 induces IL1β and IL18 secretion in inflammatory microglia via the NLRP3 inflammasome, which was supported by our data that BzATP-induced release of IL1β and IL18 was completely abolished in LPS-primed P2X7^−/−^ microglia. To pharmacologically confirm our findings and gain a broader view of secretome mediated by P2X7, we collected cell culture supernatants from LPS alone or LPS plus IFNγ primed microglia in the presence or absence of A-804598 prior to BzATP treatment, and then measured the levels of 38 secreted signaling proteins using commercially available multiplex antibody-based immunoassays. Thirty factors were detectable with the assay. Microglia under various conditions showed a distinct profile of secreted signaling factors, as visualized by unbiased cluster analysis (Fig. 7a). Interestingly, we observed that IFNγ suppressed many LPS-induced pro-inflammatory changes in mouse microglia in our selected secretion panel (Fig. 7a). Indeed, we detected a significant amount of pro-inflammatory IL1β in the supernatants from microglia primed with LPS or LPS plus IFNγ in the presence with BzATP, but this was not the case for A-804598 pre-treated microglia (Fig. 7b). In addition, A-804598 appeared not to significantly affect the secretion of the other factors except for IL1α in our cytokine panel, indicating the specific role of P2X7 signaling in mediating the release of IL1 family cytokines (Fig. 7a, c). For further validation, we compared the secretion of four inflammatory cytokines, IL1α, IL1β, TNFα, and IL6, under different treatment conditions. Figure 7b, c showed that the release of IL1α and IL1β was greatly impaired by A-804598 in BzATP stimulated inflammatory microglia, while the release of TNFα and IL6 was not significantly affected (Fig. 7d, e). In summary, our data characterized the unique P2X7 secretome in microglia and revealed that IL1α and IL1β were the only cytokines regulated by P2X7 in our selected panel.

### AKT and ERK pathways are downstream of BzATP-induced P2X7 activation

**Fig. 8:**
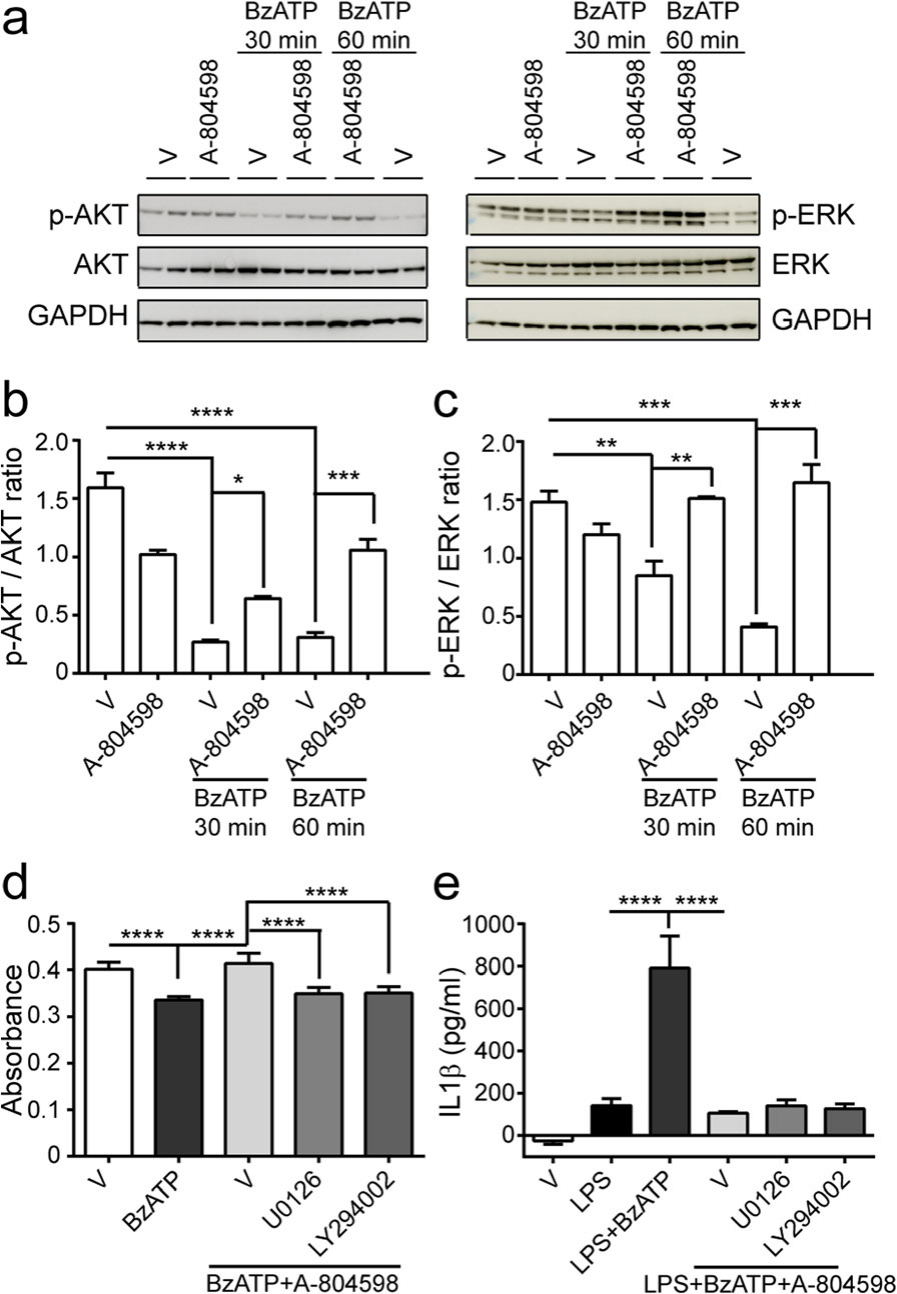
AKT and ERK pathways are involved in BzATP-induced P2X7 activation. **a**–**c** BzATP was applied to microglial culture for 30 min or 1 h in the absence or presence of A-804598. **a** Western blots were probed with antibodies against p-AKT and p-ERK. **b**, **c** Quantification of p-AKT (**b**) and p-ERK (**c**) signal intensity normalized to AKT and ERK, respectively. **d** Microglial cell death was assessed with the treatments of 50 μM U0126 or 1 μM LY294002 in the presence of 10 μM A-804598 for 1 h prior to 380 μM BzATP stimulation for additional 4.5 h. **e** Microglia were primed by 100 ng/ml LPS for 22 h followed by 50 μM U0126 or 1 μM LY294002 treatment in the presence of 10 μM A-804598 1 h before 380 μM BzATP stimulation for additional 2 h. IL1β release was then conducted by ELISA. Experiment was repeated at least twice independently. One-way ANOVA followed by Tukey’s post hoc test. *P < 0.05; **P < 0.01; ***P < 0.001; ****P < 0.0001

P2X7 activation stimulates AKT phosphorylation in astrocytes and ERK phosphorylation in renal fibroblasts [29, 30]. However, the signaling mechanisms underlying P2X7 activation in microglia is less defined. Hence, we further verify if AKT and ERK pathways contribute to P2X7 activation in microglia. We performed time-course studies with BzATP in the presence or absence of A-804598. Figure 8a revealed a rapid dephosphorylation of AKT and ERK within 30 min of stimulation with BzATP, which reached the peak and persisted for 1 h. In contrast, when we treated the cultures with A-804598 prior to stimulation with BzATP, dephosphorylation of AKT and ERK induced by BzATP was significantly suppressed in a time-dependent manner (Fig. 8a–c). To verify involvement of the ERK and AKT pathways in BzATP-induced microglial cell death and IL1β release, we treated microglia with ERK pathway inhibitor U0126 or AKT pathway inhibitor LY294002 1 h before BzATP stimulation in the presence of A-804598. Results showed that U0126 and LY294002 significantly blocked the rescue effects of A-804598 in BzATP-induced microglial cell death (Fig. 8d). However, suppression of IL1β release by A-804598 was not influenced by either U0126 or LY294002 (Fig. 8e). Therefore, our data indicate P2X7 activation is coupled to AKT and ERK signaling, which mediates BzATP-induced microglial cell death but not IL1β release.

## Discussion

In the current study, both genetic and pharmacological approaches demonstrate that P2X7 mediates BzATP-induced cell death and secretion of IL1 family cytokines in mouse microglia. First, we showed constitutive expression of P2X7 in mouse and human microglia. The expression of P2X7 was decreased in microglia following LPS priming at all tested time points; in the presence of LPS and IFNγ, we observed a transient increase followed by a decrease of *P2rx7* mRNA level. Moreover, BzATP stimulation led to cell death and robust release of IL1β and IL18 in WT microglia, while this effect was inhibited in P2X7^−/−^ microglia. To validate that P2X7 mediates microglial cell death and cytokine release, the highly selective P2X7 antagonist A-804598 was used to block BzATP-induced cell death and secretion of IL1β and IL1α in microglia. Last, we verified P2X7 activation is linked to AKT and ERK pathways which contribute to cell death but not the production of IL1 family cytokines via P2X7 in microglia.

P2X7 was expressed in microglia (Fig. 1), and its mRNA level was modestly regulated in inflammatory microglia (Fig. 2). However, it is the functional changes of P2X7 activation that plays a critical role during neuroinflammation. Under normal physiology, P2X7 is not functionally engaged since it takes high micromolar concentrations of ATP to activate P2X7, and extracellular ATP is rapidly degraded [7]. On the other hand, during neuroinflammation under cellular stress and necrosis, intracellular ATP (often millimolar) is dumped in the extracellular space that is sufficient to activate P2X7. Thus, functional consequences of P2X7 activation more than expression changes are important pathological drives of disease process.

P2X7 is expressed predominantly in cells of hematopoietic lineage, and its function is mixed by the presence of N- and C-terminal splice variants. There are several strains of P2X7^−/−^ mice generated by inserting a lacZ and a neomycin cassette into exon 1 (Glaxo) [31], or by inserting a neomycin cassette into exon 13 (Pfizer) [18, 32], or by knocking-in of human *P2rx7* cDNA to the mouse *P2rx7* locus [14], or by inserting shRNA vectors [33]. In the current study, we used microglia from Pfizer P2X7^−/−^ mice, which showed low levels of C-terminal truncated splice variants as detected by reverse transcription PCR using primers specific for various *P2rx7* transcripts (Fig. 3a). This finding is reminiscent of Masin’s report [28]. The reduced function of C-terminal truncated splice variants in P2X7^−/−^ mouse may partly explain the difference in cytotoxicity and cytokine secretion profiles between P2X7^−/−^ microglia and microglia exposed to P2X7 antagonist A-804598.

P2X7 is mainly responsible for ATP-induced cell death not only in immune cells such as macrophage [22], dendritic cells [34], mast cells, and lymphocytes [23] but also in cancer cells [35] and neural stem cells [36]. Our data are in line with these reports and expand the role of P2X7 in microglial cell death induced by BzATP, a more potent agonist for P2X7 than ATP (Figs. 4 and 6). In addition to quiescent microglia, BzATP also caused cell death in LPS-primed microglia, suggesting that P2X7 in activated microglia is still sufficient to function upon BzATP. Compared with knockout of P2X7, A-804598 did not completely abrogate BzATP-induced cell death. This may be explained by the reversibility of A-804598 binding to P2X7 [37]. Moreover, P2X7 is known to mediate cell death through either apoptosis [34] or necrosis [23]. Nevertheless, whether apoptosis or necrosis is the predominant mechanism for P2X7-dependent microglial cell death remains to be determined.

Secretion of IL1 cytokine family needs two steps which includes activation of both toll-like receptors and inflammasome. P2X7 is one of the most potent activators of NLRP3 and makes NLRP3 inflammasome sensitive to extracellular ATP. In our study, we confirmed P2X7-dependent release of IL1β and IL18 in pro-inflammatory microglia responding to BzATP (Fig. 5). Furthermore, our 38-cytokine multiplex results exhibited that only IL1β and IL1α were suppressed in pro-inflammatory microglia with BzATP stimulation when exposed to A-804598 (Fig. 7), which supports the major player of P2X7 in the production of IL1 family cytokines. Interestingly, we found that LPS alone induced release of IL1β in WT microglia which was not P2X7 dependent as a similar release was observed in the P2X7^−/−^ microglia (Fig. 5a, b). Contrary to this, IL18 release was entirely dependent of P2X7 activation because knockout of P2X7 completely blocked LPS plus BzATP-induced IL18 release (Fig. 5c, d). More intriguingly, we observed that LPS plus IFNγ-primed WT cells did not elicit IL1β or IL18 release as high as LPS alone-primed WT cells (Fig. 5), indicating that the P2X7 signaling arm may be modified by priming with different cytokine stimuli.

AKT and ERK pathways are involved in a variety of biological events, such as cell differentiation, cell survival, cell cycle, and protein synthesis. P2X7 activation induces AKT phosphorylation in rat cortical astrocytes [29], while ERK cascade has been identified as a key signaling pathway for P2X7-induced death of renal fibroblasts [30]. In contrast, we found de-phosphorylation of ERK and AKT upon P2X7 activation in response to BzATP in microglia (Fig. 8), which may be due to the context-dependent manner of these pathways such as in different cell types [38]. Furthermore, we verified that the ERK and AKT pathways mediated BzATP-induced microglia cell death but not IL1β release, confirming that P2X7 is coupled to AKT and ERK activation.

Primary cultures provide sound in vitro models for studying molecular mechanism and directly testing the effect of compounds on microglial activation and function. However, as any in vitro models, culture conditions may not reflect exactly the in vivo environment, which possibly alter astrocyte and microglial gene expression as compared them to their naïve counterparts in the brain [39]. These changes are most likely attributed to the exposure to serum with different components and concentrations from those in the brain, the absence of neurons and other cell types in culture, as well as the altered cell type ratios from which they are exposed in vivo. Therefore, gene expression and phenotypes of microglia and astrocytes from in vitro studies should be carefully considered when translated to an in vivo setting. As such, further studies for P2X7 function in microglia in vivo are critical for the development of effective therapies for neurological diseases.

## Conclusions

Our data demonstrate that P2X7, as a modulator of neuroinflammation, plays a significant role in mediating microglial cell death and cytokine release, which may be coupled to AKT and ERK pathways. These findings might provide potential implications for the identification and development of novel therapies against neuroinflammation-associated disorders.

## Additional files

Additional file 1: Concentration and time-dependent responses of microglia in cell death upon BzATP stimulation. Microglial cell death was measured at indicated time points with different concentrations of BzATP stimulation. Data were normalized to 0 h. Two-way ANOVA followed by Tukey’s post hoc test. Different concentrations of BzATP was compared to 0 μM. ****P < 0.0001. (DOCX 83 kb)

Additional file 2: Concentration and time-dependent responses of microglia in IL1β release upon LPS plus BzATP stimulation. a IL1β secretion was detected in LPS-primed microglia with different concentrations of BzATP for 2 h. b IL1β secretion was detected in LPS-primed microglia with 380 μM BzATP for different time points. One-way ANOVA followed by Tukey’s post hoc test. ***P < 0.001; ****P < 0.0001. (DOCX 121 kb)

## Abbreviations

Arg1: Arginase 1
BzATP: 3′-O-(4-Benzoyl) benzoyl adenosine 5′-triphosphate
CCK-8: Cell counting kit-8
CCL2: Chemokine (C-C motif) ligand 2
ELISA: Enzyme-linked immunosorbent assay
IFNγ: Interferonγ
IL: Interleukin
LPS: Lipopolysaccharide
MACS: Antigen-antibody-mediated magnetic cell-sorting
Nos2: Nitric oxide synthase 2
RNA-seq: RNA-sequencing
TNFα: Tumor necrosis factor α
WT: Wild type
