# Supplementary material for: The role of microglial P2X7: modulation of cell death and cytokine release
Source: J Neuroinflammation. 2017 Jul 17;14:135. doi: 10.1186/s12974-017-0904-8 (PMC5513370; doi:10.1186/s12974-017-0904-8)
Supplement: Supplementary file 1 — Concentration and time-dependent responses of microglia in cell death upon BzATP stimulation. Microglial cell death was measured at indicated time points with different concentrations of BzATP stimulation. Data were normalized to 0 h. Two-way ANOVA followed by Tukey’s post hoc test. Different concentrations of BzATP was compared to 0 μM. ****P < 0.0001. (DOCX 83 kb) [file 12974_2017_904_MOESM1_ESM.docx]

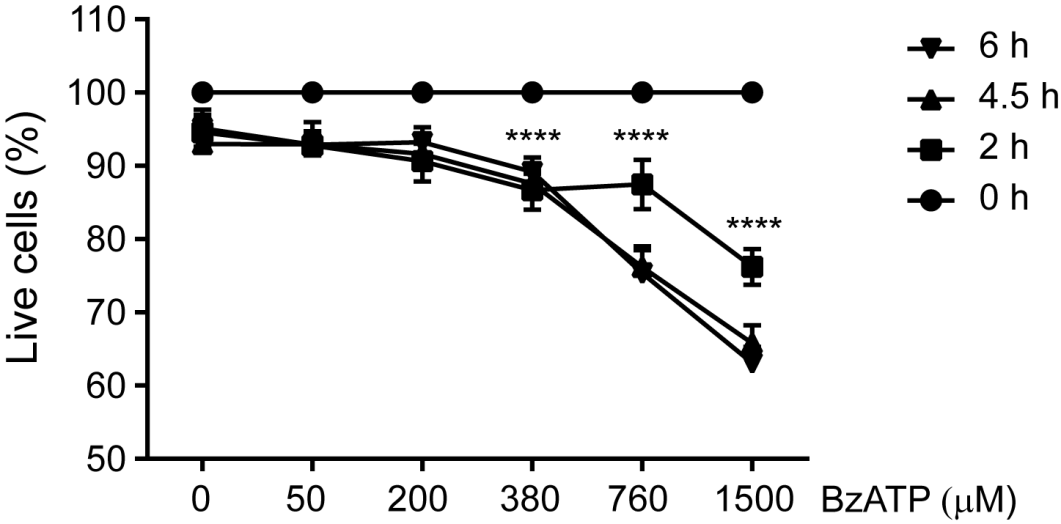


**Additional file 1: Concentration and time-dependent responses of microglia in cell death upon BzATP stimulation.** Microglial cell death was measured at indicated time points with different concentrations of BzATP stimulation. Data were normalized to 0 hour. Two-way ANOVA followed by Tukey's post hoc test. Different concentrations of BzATP was compared to 0 μM. ****, *P* < 0.0001.
