# Supplementary material for: The role of microglial P2X7: modulation of cell death and cytokine release
Source: J Neuroinflammation. 2017 Jul 17;14:135. doi: 10.1186/s12974-017-0904-8 (PMC5513370; doi:10.1186/s12974-017-0904-8)
Supplement: Supplementary file 2 — Concentration and time-dependent responses of microglia in IL1β release upon LPS plus BzATP stimulation. a IL1β secretion was detected in LPS-primed microglia with different concentrations of BzATP for 2 h. b IL1β secretion was detected in LPS-primed microglia with 380 μM BzATP for different time points. One-way ANOVA followed by Tukey’s post hoc test. ***P < 0.001; ****P < 0.0001. (DOCX 121 kb) [file 12974_2017_904_MOESM2_ESM.docx]

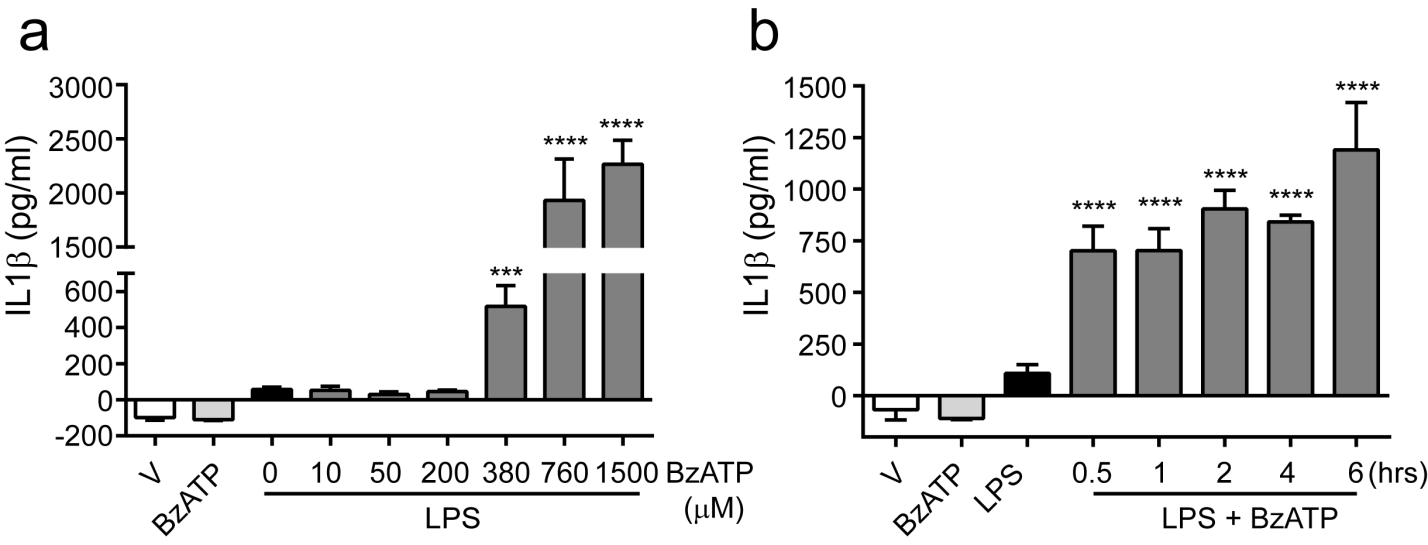


**Additional file 2:** **Concentration and time-dependent responses of microglia in IL1β release upon LPS plus BzATP stimulation. a** IL1β secretion was detected in LPS primed microglia with different concentrations of BzATP for 2 hours. **b** IL1β secretion was detected in LPS primed microglia with 380 μM BzATP for different time points. One-way ANOVA followed by Tukey's post hoc test. *** *P* < 0.001; ****, *P* < 0.0001.
